# Antihypertensive treatments and risks of lung Cancer: a large population-based cohort study in Hong Kong

**DOI:** 10.1186/s12885-021-08971-6

**Published:** 2021-11-11

**Authors:** Jinhui Li, Amy S. M. Lam, Sarah T. Y. Yau, Karen K. L. Yiu, Kelvin K. F. Tsoi

**Affiliations:** 1 JC School of Public Health and Primary Care, The Chinese University of Hong Kong, Shatin, Hong Kong; 2 Department of Medicine and Therapeutics, Faculty of Medicine, The Chinese University of Hong Kong, Shatin, Hong Kong; 3 SH Big Data Decision Analytics Research Centre, The Chinese University of Hong Kong, Shatin, Hong Kong

## Abstract

**Background:**

There is a growing concern that the use of anti-hypertensives may be associated with an increased risk of cancer, but it remains uncertain for the association between anti-hypertensives and lung cancer risk, as well as their interaction with aspirin in chemoprotective effects.

**Methods:**

The goal of this study is to assess the association between anti-hypertensives use and the risk of lung cancer, as well as the chemopreventive impacts from the combination usage of aspirin and anti-hypertensives. A retrospective cohort study was conducted based on all the public hospital electronic medical records in Hong Kong. Patients with prescription records of anti-hypertensives (ACEi/ARB, CCB, *β*-blocker,*α*-blocker) and/or aspirin were included as the exposure groups. Using the Cox proportional hazards model with inverse probability weighting, we estimated hazard ratios (HRs) with 95% confidence intervals (CIs) for lung cancer risk from anti-hypertensives usage or combination usage of aspirin with anti-hypertensives. The likelihood ratio test and interaction model were adopted for exploring the interaction effects with aspirin.

**Results:**

A total of 6592 and 84,116 lung cancer cases were identified from the groups of anti-hypertensives users and anti-hypertensives users with aspirin, respectively. The group of non-aspirin patients who received anti-hypertensives showed a significantly lower risk of lung cancer (HR: 0.63, 95% CI: 0.60–0.66), compared to those without anti-hypertensives**.** When aspirin and *α*-blocker were used simultaneously, it could lower the risk of lung cancer significantly (HR: 0.53, 95% CI: 0.34–0.84). Moreover, the lower risk of lung cancer persisted with a longer follow-up period of anti-hypertensives usage. Combination usage with aspirin in the users of ACEi/ARB, CCB, and *α*-blocker showed significant interaction effects. However, the smoking effect could not be eliminated in this analysis.

**Discussion:**

Anti-hypertensive treatment was associated with a lower risk of lung cancer, which is associated with the anti-hypertensives exposure period. The potential interaction on the chemopreventive influence from combination usage of α-blocker and aspirin might exist. More corroborations on these findings are needed to focus on the different settings in future studies.

**Supplementary Information:**

The online version contains supplementary material available at 10.1186/s12885-021-08971-6.

## Introduction

Lung cancer has been the leading cause of cancer death worldwide, accounting for an estimated 1.76 million cases in 2018 [1]. In Asia, there was a steady rise in the lung cancer incidence for the past decade, accounting for more than half of the world’s lung cancer cases [2]. In Hong Kong, due to the aging population and advancing technology in disease diagnosis over the past 40 years, lung cancer has been the second most common cancer in men and the third most common in women [3]. Multiple risk factors could induce lung cancer carcinogenesis, such as smoking, alcohol, air pollution, and diet [4]. Given the significant disease burden from high fatality and poor prognosis [5], more preventative approaches for lung cancer are still needed. The persisting grim lung cancer morbidity and mortality figures motivate severe therapies such as chemoprevention to be conducted. In our previous cohort study, it was identified that long-term low-dose aspirin usage could reduce the lung cancer risk among Hong Kong patients [6]. A Korean nationwide retrospective cohort study also reported that a 5-year low-dose aspirin regimen was associated with lower risk of lung cancer [7]. However, the long-term usage of antihypertensives showed an inconsistent association with lung cancer. One study indicated that the use of ACEis was associated with an increased risk of lung cancer [8], while another demonstrated no significant cancer risk from long-term use of antihypertensives [9].

The antihypertensives include angiotensin I-converting enzyme inhibitors (ACEis), angiotensin II receptor blockers (ARBs), calcium channel blockers (CCBs), diuretics, *Alpha*-adrenergic receptor blockers (*α*-blockers), and *Beta*-adrenergic receptor blockers (*β*-blockers) [10]. In Hong Kong, it is reported that hypertension prevalence was 27.7% among those between 15 and 84 years old. The hypertension rate was about 65% in the population aged between 65 years and 84 years [11].. A study based on the 3-year Hong Kong primary care clinics data summarized that the CCBs were the most commonly used (49%), followed by *β*-blockers and ACEi (46 and 19% respectively) among all the prescription anti-hypertensive agents [12]. Considering that hypertension and cancers share some common risk factors and cardio-toxicity pathways, the association between anti-hypertensives therapy and carcinogenicity attracts more attention from researchers and the findings are still controversial.

As an aging society with an elderly population expected to double over the next 25 years, the burden of cancer and cardiovascular (CVD) in Hong Kong is predicted increase dramatically. In light of the conflicting and limited evidence from Asian population on the long-term anti-hypertensives use and lung cancer risk, a large population-based cohort study was conducted [6]. We aimed to assess if there exists an association between anti-hypertensives use and the risk of lung cancer, as well as the chemopreventive impacts from the combination usage of aspirin and anti-hypertensives.

## Study design and methods

This is a retrospective cohort study which enrolled long-term aspirin and antihypertensive drugs users with 10 years of follow-up within electronic medical records in all the public hospitals of Hong Kong. The data records could represent the whole population of aspirin usage due to the reason that the Hong Kong Hospital Authority from Hong Kong government supports the long-term care for citizens who have chronic diseases with little expense for patients. Thus, the database in this study is representative of the population that had chronic diseases while maintaining aspirin and anti-hypertensives usage.

In addition, our study followed the standard guidelines of Strengthening the Reporting of Observational Studies in Epidemiology (STROBE). All medical records were obtained anonymously and patients were assigned anonymous identification code as required by the policy of Hong Kong Hospital Authority from Hong Kong government. As a result, the consent to participate was not applicable and the informed consent was waived, by the ethical approval reached from the Joint Chinese University of Hong Kong - New Territories East Cluster Clinical Research Ethics Committee (CREC ref. no.:2014.461).

### Data source

From the Hospital Authority Clinical Data Repository, among the 4,564,100 eligible study participants who had ever used any public healthcare service between 2000 and 2004, subjects aged over 18 years old with aspirin/anti-hypertensives prescription and the age-and-gender-matched control group with non-aspirin at the ratio of 1:2 were included in our original study [6] (*N =* 602,936). Participants who were diagnosed with cancer according to the ICD codes were extracted from Hong Kong electronic health system. Individuals with prevalent cancer at the start of follow up were excluded, but individuals with cancer found in different locations in the follow-up duration were included in the study. Moreover, all the patients’ data extraction for academic institutions are under the strict supervision and regulation for protection of privacy.

### Medication use

Participant follow-up time was measured from the time of cohort entry, until the date of death or end of 2013, whichever came first. The medication and disease diagnosis records were kept tracking for each participant. Index date was defined as the first prescription record of aspirin or anti-hypertensives drugs. The durations of aspirin and anti-hypertensives prescription histories were computed for each participant by calculating the first and last prescriptions time. Among them, patients who had taken aspirin and anti-hypertensive drugs for less than 6 months were excluded. If a patient suspended the medication prescription temporarily, the gap period of no aspirin/anti-hypertensives usage was also calculated. Generally, the aspirin prescription in Hong Kong is mainly to prevent cardiovascular and cerebrovascular diseases, so patients with comorbidities of CVDs, including ischemic heart diseases (IHD) (ICD-9: 410–414), stroke (ICD-9:430, 431, 433, 434, 436), and other cerebrovascular diseases (ICD-9: 430–438), were identified from the electronic health system. Additionally, we also documented the other medications usage including antiplatelet agents (apart from aspirin), non-steroidal anti-inflammatory drugs (NSAID), anticoagulant (warfarin and direct oral anticoagulants) and other antisecretory agents such as proton pump inhibitor (PPI) or H2-antagonist (H2B) [6]. As *α*-blocker also belongs to the main therapy in men with lower urinary tract symptom, the only *α*-blocker drugs intake group just included the male patients in our study. Moreover, ACEi and ARB shared the similar mechanism in the renin-angiotensin-aldosterone system (RAAS) pathway that is widely implicated in diabetes (DM), hypertension, and heart failure, so they were analysed as a group of ACEi/ARB. The usage of diuretics only was excluded as it was also widely used for other symptoms such as oedema due to different underlying causes.

### Statistical analyses

The frequencies with percentages for categorical variables, medians with interquartile ranges (IQR), or means with standard deviations (SD) for continuous variables were calculated during the whole cohort follow-up period. We adopted the Cox proportional hazards regression models with inverse probability (IP) to assess the hazard ratios (HR) with 95% confidence intervals (CIs) for the association between different medication usages of 1) aspirin usage, 2) aspirin and anti-hypertensives combination usage, and lung cancer risks. The different timescales for the follow-up time points included the years of 2006, 2009, and 2013. IP weighting was used to adjust for confounding factors including NSAID, H2 blocker, anticoagulant, antiplatelet, PPI, statin, as drug usages can indirectly reflect comorbidities of patients. Then the likelihood ratio test was adopted to compare the additive model and the interaction model, and the interaction effects with the cross-classification by anti-hypertensives and aspirin usage were explored. In addition, follow-up and subgroup analyses were performed by different follow-up period, gender, DM, and age groups. Sensitivity analysis with different drugs exposure period were also conducted to evaluate the robustness of findings. All models were adjusted for the following variables measured at cohort entry: age, sex, comorbidities, and other medications. *P*-value < 0.05 was adopted to define statistical significance. All the analyses were performed in the R package version 3.6.1.

## Results

### Baseline characteristics

**Fig. 1:**
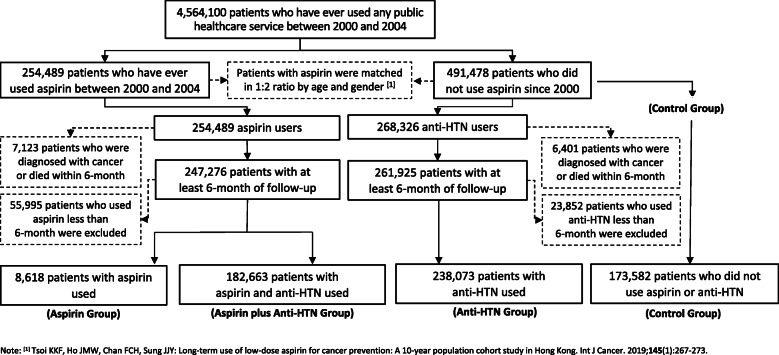
Flowchart of the study to show the number of participants after each exclusion criteria

Among all the 4,564,100 patients who came to visit the clinics or hospitals under the Hospital Authority internal network during the period of 2000–2004, there were 745,967 subjects who were identified as aspirin users and its matched control group. After applying the exclusion criteria, the final total sample size for this study was 602,936 (Fig. 1). In detail, 13,614 patients were removed as they were diagnosed with cancer or died within 6 months, and 79,847 were excluded for the reason that the duration of drugs usage were less than 6 months. There were 8618 patients who had the prescription medication of aspirin, 238,073 (39.5%) who had the prescription medication of anti-hypertensives, 182,663 (30.3%) who had both the aspirin and anti-hypertensives, and 173,582 (28.8%) as control group.

For the participants enrolled in the study, the mean age was 67.5 (SD = 12.0) years old. Table 1 shows the characteristics of all the participants in the study. The majority of subjects used more than one type of anti-hypertensives in the anti-hypertensives group (77.6%) and aspirin & anti-hypertensives group (91.3%). CCB and *β*-blocker were the most popular single anti-hypertensive drugs for the group of anti-hypertensives (9.6%) and aspirin & anti-hypertensives separately (3.3%) respectively. Additional characteristics for the group of anti-hypertensives users and non-anti-hypertensives users are also shown in Table S1 of the Supplementary section.

**Table 1:** Characteristics of the Study Participants

	Anti-hypertensives (***n*** = 238,073)	Aspirin + Anti-hypertensives (***n*** = 182,663)	Aspirin (***n*** = 8618)	Control (***n*** = 173,582)
**Age**				
< 50	4951 (2.1%)	10,663 (5.8%)	1973 (22.9%)	41,084 (23.7%)
50–64	41,449 (17.4%)	44,771 (24.5%)	2119 (24.6%)	50,773 (29.3%)
65–79	131,267 (55.1%)	95,206 (52.1%)	2785 (32.3%)	61,588 (35.5%)
> =80	60,406 (25.4%)	32,023 (17.5%)	1741 (20.2%)	20,137 (11.6%)
**Sex**				
Male	122,634 (51.5%)	98,878 (54.1%)	4130 (47.9%)	92,703 (53.4%)
Female	115,439 (48.5%)	83,785 (45.9%)	4488 (52.1%)	80,879 (46.4%)
**Use of anti-hypertensives**				
ACEi/ARB only	4986 (2.1%)	2140 (1.2%)	NA	NA
*α*-blocker only	11,297 (4.7%)	1041 (0.6%)	NA	NA
*β*-blocker only	7085 (3.0%)	5980 (3.3%)	NA	NA
CCB only	22,925 (9.6%)	4816 (2.6%)	NA	NA
Combined treatment	184,725 (77.6%)	166,754 (91.3%)	NA	NA
**Mean Duration**				
Aspirin	NA	8.0 years	5.5 years	NA
Anti-hypertensives	7.0 years	8.7 years	NA	NA
ACEi/ARB	4.9 years	5.8 years	NA	NA
*α*-blocker	5.0 years	4.9 years	NA	NA
*β*-blocker	5.4 years	7.0 years	NA	NA
CCB	4.7 years	5.7 years	NA	NA
**Other Medication Use**				
H2 blocker	142,158 (59.7%)	144,348 (79.0%)	5754 (66.8%)	65,753 (37.9%)
Statin	48,447 (20.3%)	98,280 (53.8%)	2255 (26.2%)	4699 (2.7%)
NSAID	130,894 (54.9%)	93,200 (51.0%)	3965 (46.0%)	73,799 (42.5%)
Anti-coagulant	13,483 (5.7%)	59,129 (32.4%)	720 (8.4%)	2543 (1.5%)
Anti-platelet	4906 (2.1%)	56,305 (30.8%)	1024 (11.9%)	600 (0.3%)
PPI	63,488 (26.7%)	87,100 (47.7%)	2383 (27.7%)	22,436 (12.9%)
**Disease Diagnosis**				
IHD	8899 (3.7%)	89,185 (48.8%)	1074 (12.5%)	1809 (1.0%)
Stroke	8506 (3.6%)	52,101 (28.5%)	2332 (27.1%)	1271 (0.7%)
IHD or Stroke	16,569 (7.0%)	121,862 (66.7%)	3250 (37.7%)	3024 (1.7%)
Diabetes mellitus	67,914 (28.5%)	73,784 (40.4%)	1230 (14.3%)	8551 (4.9%)
**No. of Lung Cancer**	6592 (2.8%)	4470 (2.4%)	216 (2.5%)	6407 (3.7%)
**No. of Death**	74,424 (31.3%)	84,116 (46.0%)	3253 (37.7%)	36,564 (21.1%)
**Duration of Follow-up**^**#**^	91 months (47.5)	115 months (46.6)	105 months (51.6)	142 months (49.3)

Note: ^#^ mean and standard deviation (SD)

Abbreviations: ACEi, angiotensin-converting enzyme inhibitor; ARB, angiotensin II receptor blocker; CCB, calcium channel blocker; H2, histamine-2 receptor; NSAID, non-steroidal anti-inflammatory drug; PPI, proton-pump inhibitor; IHD, ischemic heart disease; NA, not applicable

### Lung cancer risk analysis

Overall, the lung cancer incidences for different group were displayed in Table 1. The lung cancer incidence of the control group was the highest with 6407 cases (3.7%), followed by the groups of only anti-hypertensives users (*n* = 6592; 2.8%), only aspirin users (*n* = 216; 2.5%), and combination of anti-hypertensives and aspirin users (*n* = 4470; 2.4%). Table 2 showed the main analysis on the lung cancer risk for the groups of only anti-hypertensives users and combination of aspirin/anti-hypertensives users separately. Compared to the control groups, anti-hypertensives usage was associated with an overall of 37% lower risk of lung cancer (HR = 0.63; 95% CI = 0.60–0.66), and combination usage of anti-hypertensives and aspirin could also reduce lung cancer risk by 36% (HR = 0.64; 95% CI = 0.60–0.68). As for the specific type of anti-hypertensives drugs, ACEi/ARB (HR = 0.45; 95% CI = 0.32–0.62), *β*-blocker (HR = 0.79; 95% CI = 0.65–0.96), and *α*-blocker (HR = 0.61; 95% CI = 0.48–0.77) were associated with a statistically significant lower risk of lung cancer.

**Table 2:** Lung cancer risk for only antihypertensive groups and combination aspirin groups*

Exposure	Anti-hypertensives					Anti-hypertensives + Aspirin				
	N	Cases	HR	95% CI	*p*	N	Cases	HR	95% CI	*p*
All Anti-hypertensives	238,073	6592 (Control: 3192)	0.63	0.60–0.66	< 0.001	182,663	4470 (Control:3215)	0.64	0.60–0.68	< 0.001
ACEi/ARB	4986	78 (Control: 655)	0.45	0.32–0.62	< 0.001	2140	67 (Control: 637)	0.71	0.48–1.05	0.090
*β*-blocker	7085	158 (Control: 614)	0.79	0.65–0.96	0.017	5980	143 (Control: 687)	0.81	0.62–1.05	0.115
CCB	22,925	570 (Control: 618)	0.89	0.73–1.08	0.222	4816	169 (Control: 664)	0.82	0.64–1.06	0.129
*α*-blocker	11,297	438 (Control: 438)	0.61	0.48–0.77	< 0.001	1041	46 (Control: 454)	0.53	0.34–0.84	0.007

Note: *Ref. group: Control; Age, sex, comorbidities, and medications were adjusted in the model;

Abbreviations: N, number of participants; HR, hazard ratio; CI, confidence interval; ACEi, angiotensin-converting enzyme inhibitor; ARB, angiotensin II receptor blocker; CCB, calcium channel blocker

### Interactive effects analysis

Interactive model was adopted. After the adjustment of age, sex, comorbidities, and medications, the significant interaction effects persisted among the combination treatment of ACEi/ARB with aspirin, CCB with aspirin, as well as *α*-blocker with aspirin (*P* < 0.001, Table S2). When *α*-blocker was used alone, it did not lower the risk of lung cancer significantly, while when it was used with aspirin together, the lung cancer risk was lowered by 47% (HR = 0.53; 95% CI = 0.34–0.84) with significant interaction (*P* = 0.008). However, no significant interaction effects were identified between all anti-hypertensives and aspirin usage (*P* = 0.090).

### Follow-up analysis

There are three follow-up time points in this study (2006, 2009, and 2013) to evaluate the impacts of the duration of medication usage to the lung cancer risk (Table 3). The patients who used anti-hypertensives showed consistent attenuated risk of lung cancer among all the follow-up periods (HR = 0.57, 95% CI = 0.53–0.60; HR = 0.60, 95% CI = 0.57–0.63; HR = 0.63, 95% CI = 0.60–0.66; for up to 7, 10, and 14 years, respectively). As Table 3 showed, it was found that longer cumulative duration of ACEi/ARB and *α*-blocker were associated with a solidly lower risk of lung cancer. With the follow-up period up to 7, 10, and 14 years, the HRs of lung cancer for single ACEi/ARB users were 0.55 (95%CI: 0.36–0.83), 0.53 (95% CI: 0.36–0.79), and 0.45 (95%CI: 0.32–0.62); and the HRs for *α*-blocker users were 0.70 (95%CI: 0.57–0.86), 0.68 (95% CI: 0.55–0.84), and 0.61 (95%CI: 0.48–0.77) respectively. When the aspirin was given concurrently, lower lung cancer risk remained for the users with *α*-blocker and aspirin medications for all the three follow-up periods. Concurrent use of *α*-blocker and aspirin lowered lung cancer risk by 57% (95%CI: 0.24–0.78), 59% (95%CI: 0.25–0.69), and 47% (95%CI: 0.34–0.84) for the follow-up period up to 7, 10, and 14 years respectively. Moreover, the trend test indicated that further reduced risk of lung cancer with longer use of anti-hypertensive drugs (*P* < 0.001, Table S3).

**Table 3:** Lung cancer risk of antihypertensive groups and combination of aspirin groups at different follow-up time points*

Exposure of Aspirin	End of 2006			End of 2009			End of 2013		
	< 7 years			< 10 years			< 14 years		
	HR	95% CI	***p***	HR	95% CI	***p***	HR	95% CI	***p***
**All Anti-hypertensives**									
Aspirin: NO	**0.57**	**0.53–0.60**	**< 0.001**	**0.60**	**0.57–0.63**	**< 0.001**	**0.63**	**0.60–0.66**	**< 0.001**
Aspirin: YES	**0.46**	**0.42–0.50**	**< 0.001**	**0.56**	**0.52–0.60**	**< 0.001**	**0.64**	**0.60–0.68**	**< 0.001**
**ACEi/ARB**									
Aspirin: NO	**0.55**	**0.36–0.83**	**0.005**	**0.53**	**0.36–0.79**	**0.001**	**0.45**	**0.32–0.62**	**< 0.001**
Aspirin: YES	0.63	0.38–1.02	0.060	0.67	0.44–1.01	0.055	0.71	0.48–1.05	0.090
***α*-blocker**									
Aspirin: NO	**0.70**	**0.57–0.86**	**< 0.001**	**0.68**	**0.55–0.84**	**< 0.001**	**0.61**	**0.48–0.77**	**< 0.001**
Aspirin: YES	**0.43**	**0.24–0.78**	**0.005**	**0.41**	**0.25–0.69**	**< 0.001**	**0.53**	**0.34–0.84**	**0.007**
***β*-blocker**									
Aspirin: NO	0.98	0.76–1.26	0.879	0.90	0.72–1.11	0.326	**0.79**	**0.65–0.96**	**0.017**
Aspirin: YES	0.87	0.61–1.22	0.407	0.76	0.56–1.03	0.074	0.81	0.62–1.05	0.115
**CCB**									
Aspirin: NO	1.05	0.87–1.27	0.608	1.02	0.86–1.21	0.808	0.89	0.73–1.08	0.222
Aspirin: YES	**0.67**	**0.48–0.94**	**0.021**	0.79	0.60–1.04	0.093	0.82	0.64–1.06	0.129

Note: * Ref. group: Control; Age, sex, comorbidities, and medications were adjusted in the model;

Abbreviations: HR, hazard ratio; CI, confidence interval; ACEi, angiotensin-converting enzyme inhibitor; ARB, angiotensin II receptor blocker; CCB, calcium channel blocker

### Subgroup analysis

*Age* significantly affected lung cancer risk (Table S4 - S6). For older patients who were over 65 years old, the use of anti-hypertensives drugs was associated with a significantly lower lung cancer risk (HR = 0.65, 95%CI:0.61,0.68). In particular, only use of ACEi/ARB or *α*-blocker demonstrated effects on lowering lung cancer risk (*P* < 0.001) among the patients who were older than 65 years old. Use of ACEi/ARB showed to have lower lung cancer risk by 56% (95% CI: 0.29, 0.67), while use of *α*-blocker reduced lung cancer risk by 39% (95%CI: 0.48, 0.77) among male patients. There was a significantly lower risk of lung cancer with anti-hypertensives usage regardless of DM status (Table S6). Among the patients who had DM, combination usage of anti-hypertensives and aspirin could decrease the lung cancer risk significantly(*P* < 0.001). Among the patients without DM, significantly lower lung cancer risk was observed with the use of ACEi/ARB or *α*-blocker (*P* < 0.001) but not with concurrent aspirin use.

### Sensitivity analysis

We extended the minimum drugs treatment period to 1 year, 1.5 years, and 2 years from the original 6 months (Table S7). The estimated association of lung cancer and all anti-hypertensives drugs were consistent with statistical significance (*P* < 0.001). For these different treatment durations, the estimated HR of lung cancer were 0.64 (95%CI: 0.61–0.68), 0.67 (95%CI: 0.64–0.71), and 0.70 (95%CI: 0.66–0.74), which were non-significant statistically difference from the overall estimates in the main analysis. When combination usage of anti-hypertensives and aspirin was defined with at least one year use, it could reduce 33% lung cancer risk (HR = 0.67; 95% CI = 0.62–0.71) which is also comparable to the main result with six-month exposure.

## Discussion

From this large retrospective cohort study among Chinese population, neither the single anti-hypertensives treatment nor aspirin combination usage was observed to have excess of lung cancer risk. To the best of our knowledge, this is the first study to explore the lung cancer risk from combination usage of aspirin and anti-hypertensives.

A network meta-analysis which included 70 randomised controlled trials reported no significant increased risk of cancer with the ACEi/ARB usage [13]. Moreover, long-term (> 7.5 years) use of anti-hypertensives would not promote or initiate cancer, in which lung cancer was one of the most common cancers in the Saskatchewan Health databases [9, 14], and the dose-response relationship for lung cancer were not identified [9]. The chemopreventive effects of anti-hypertensives for different types of cancer are still uncertain, but our findings in a Chinese cohort from Hong Kong are comparable with other studies that explored the association between anti-hypertensive medications and the risk of lung cancer [9, 13, 14]. Our findings also showed a comparable conclusion with a previous cohort study based on the UK general practice research database, which reported that ARB exposure would reduce the risk of lung cancer (RR = 0.87, 95%CI = 0.75–0.94) [14]. Moreover, a cohort study exploring the association between ACEi and lung cancer among the 22,384 Chinese population [15]. In this study, they found that ACEi use was associated with an increased risk of lung cancer compared with ARB use. Patients using ARBs have a significantly lower risk of lung cancer than non-ARB users. In addition, there is another study investigated the anti-hypertensives usage and survival rates for a few cancers based on the Shanghai Women’s Study (1996–2000) and Shanghai men’s Study (2002–2006) in Shanghai, China. It is identified that better Overall Survival (OS) was found among users of ARBs or CCBs in lung cancer patients. But the association was insignificant after fully adjustment of multiple confounding factors [16]. As we used ACEi/ARB medication in our study and found that ACEi/ARB could lower 37% the risk of lung cancer, more exploration on the only medication usage and ACEi/ARB usage among Chinese population are still needed. Differently, there were still a few studies showing significantly increased risk of lung cancer with anti-hypertensive treatment [8, 17]. In a population-based nested case-control study from Israel, Rotshild et al reported that the gradual usage of CCBs could modestly increase the lung cancer risk, and the association was more obvious with longer duration of the antihypertensive drugs usage [17]. From our cohort study, we did not identify any significant association between CCBs treatment and lung cancer risk (*P* > 0.05). Another study including 992,061 patients who were newly treated with anti-hypertensives showed that the ACEi treatment was associated with an increased risk of lung cancer by 14% (95%CI: 7–33%) [9]. The inconsistent results might be due to the duration of the anti-hypertensives use, with relevant shorter follow-up period [13]. A study from UK General Practice Research Database with a median of 4.6 years of follow-up showed that ACEi/ARB usage can reduce the risk of lung cancer [9], it may be the reason why we found a comparable conclusion with longer duration of follow-up. Therefore, it still needs more evidence targeting the potential oncogenic mechanisms of different classes of anti-hypertensives, as well as the interaction mechanisms for the combination usage with aspirin or other majority CVD medications.

Aside from the numerous epidemiological studies addressing the association between anti-hypertensives/aspirin to the risk of cancer imitation, promotion and recurrence, more studies on molecular pathway and mechanisms were conducted to address the conflicting results from epidemiological studies [13, 18]. Until now, the plausible mechanisms for the relationship between antihypertensives and cancer risk are still not fully understood [19]. It summarized that different angiotensin I-converting enzyme genotypes and AT1 receptors blockade with an ARB stimulate tumor angiogenesis might be related to different carcinogenesis results after ACEi/ARB treatment. However, under the potential mechanism of angiogenesis, proliferation, and inflammation blockade could contributed the protective effects [19, 20]. In addition, as an essential during molecular signal transmission in cell generally [21], CCBs could potentially inhibit cell apoptosis to active carcinogenesis pathway; while it was also found that high dose of CCBs might involve the cell proliferation and calcium influx to spark the anticancer functions [21]. Moreover, a review summarized that among 164 types of anti-hypertensives included, only 75 ones showed clear genotoxicity and carcinogenicity while the remaining ones lacked of the testing results of genotoxicity and carcinogenicity assays [22]. Earlier, based on the mammalian cells and bacteria testing, it was found that anti-hypertensives agent could damage the DNA repair and elicit the carcinogenesis pathway [23]. Studies based on the experiments in vitro and in vivo reported that ARBs could implicate the renin-angiotensin system (RAAS) and then regulate the cell proliferation, angiogenesis, metastasis, and apoptosis [24, 25]. A recent study from a Taiwan team demonstrated that CCBs showed anticancer effects on the chemoresistant lung cancer cells (A549 lung adenocarcinoma chemoresistant sublines) through autophagy and apoptosis [26]. Furthermore, it was reported that aspirin usage could activate several inflammation-related critical transcription factors such as nuclear factor k-light-chain (NF-κB), oxygen species, cytokines and growth factors, and proinflammatory enzymes, which is essential factor in carcinogenesis, as well as stimulating epigenetic modifications [27]. In our study, compared to the control group, it was interesting to identify that usage of *α*-blocker and aspirin showed better chemopreventive impacts on the lung cancer risk for the patients who had no DM. The results were also consistent with the main findings from the overall cohort, while the consistent trend was not identified from other specific anti-hypertensives when they were used with aspirin synchronously. This might be due to some common molecular mechanisms in the chemotherapy pathway for the treatment of aspirin and antihypertensive, such as the enzyme cyclooxygenase (Cox) inhibition [28]. Moreover, patients with diabetes could have 2–4 folds of increased risk of dying from complications of CVD [29], which might modify the effects as well. Until now, there is still no proven mechanism on the chemotherapy of aspirin or antihypertensive in the tumorigenesis pathway, more insights for the explanation are warranted. Considering that carcinogenesis of lung cancer is a series of complicated processes including multiple genomic, epigenetic, epigenomic, proteomic and metabolomics modification that involves the interaction with environmental, nutrition, and lifestyle, more reliable evidence is warranted to explore the biological mechanism in the carcinogenesis pathway and medication metabolism.

The strengths of this study include 1) large study population in Hong Kong, 2) long follow-up period, and 3) different classes of anti-hypertensives studied. Despite such, some limitations should still need to be noticed. First, smoking status is the major confounder for the risk of lung cancer, but our hospital records did not include the history of smoking among the users of aspirin and anti-hypertensives. Previous studies demonstrated that patients with CVD may encourage to quit smoking, which would reduce their risk of lung cancer [30, 31]. Second, although we tried to control for different confounding variables in the models, including age, sex, IHD, baseline comorbidities and other chronic medication use, there were still some unviable confounders in this hospital-based cohort such as smoking with respiratory problems, family history of lung cancer. The results from this hospital-based cohort would not be able to represent the general population in Hong Kong. Additionally, the age discrepancy between the control and exposure groups might remain a limitation of this study, although the age has been adjusted. Further, as the majority of aspirin users were relatively older and the controls were matched by age to them, this study would be limited to the older part of the cohort, with a potential under-representation of younger users of Anti-hypertensives. Finally, the findings mainly focused on Chinese participants, so caution should be taken in the interpretation for other populations. Finally, there was no information on the prior exposure of anti-hypertensives among the participants, so the actual exposure duration of anti-hypertensives was unsure.

## Conclusion

In conclusion, through the large population-based retrospective cohort study in Hong Kong, the lung cancer incidence risk from long-term anti-hypertensives treatment was assessed. Anti-hypertensive medication was associated with a lower risk of lung cancer, especially for male patients or those aged over 65 years old. Similar conclusions were reached when assessing the lung cancer risk from the only ACEi/ARB and *α*-blocker usage. The potential interaction on the chemopreventive influence from combination usage of *α*-blocker and aspirin might exist. These associations appeared to attenuate with longer duration of usage. Due to the observational nature of this study, the results shall be interpreted carefully. More corroborations on these findings are needed to focus on the different settings in future studies.

## Supplementary Information

**Additional file 1.**

## Data Availability

The datasets generated during and/or analysed during the current study are not publicly available due to Data Protection Laws and Regulations in Hong Kong, but final analysing results are available from the corresponding authors on reasonable request.
