# Supplementary material for: Antihypertensive treatments and risks of lung Cancer: a large population-based cohort study in Hong Kong
Source: BMC Cancer. 2021 Nov 11;21:1202. doi: 10.1186/s12885-021-08971-6 (PMC8582182; doi:10.1186/s12885-021-08971-6)
Supplement: Supplementary file 1 — Additional file 1. [file 12885_2021_8971_MOESM1_ESM.docx]

**Table S1.** Additional Characteristics (Anti-hypertensives *vs* No Anti-hypertensives)

|  | **Anti-hypertensives**  **(n=420,463)** | **No Anti-hypertensives// Control**  **(n=182,199)** |
| --- | --- | --- |
| **Age** |  |  |
| <50 | 14,806 (3.5%) | 43,398 (23.8%) |
| 50-64 | 84,932 (20.2%) | 52,873 (29.0%) |
| 65-79 | 226,122 (53.8%) | 64,379 (35.3%) |
| >=80 | 94,603 (22.5%) | 21,549 (11.8%) |
|  |  |  |
| **Sex** |  |  |
| Male | 221,338 (52.6%) | 96,833 (53.1%) |
| Female | 199,125 (47.4%) | 85,366 (46.9%) |
|  |  |  |
| **Anti-hypertensives usage** |  |  |
| ACEi/ARB only | 7,123 (1.7%) | NA |
| Beta-blocker only | 13,055 (3.1%) | NA |
| CCB only | 27,720 (6.6%) | NA |
| Alpha-blocker only | 12,325 (2.9%) | NA |
| More than one type | 351,280 (83.5%) | NA |
|  |  |  |
| **Mean Duration of Medication usage** |  |  |
| Aspirin (among those who ever took aspirin) | 7.97 years | 5.48 years |
| Anti-hypertensives | 7.72 years | NA |
| ACEi/ARB | 5.14 years | NA |
| *β*-blocker | 6.13 years | NA |
| CCB | 4.89 years | NA |
| *α*-blocker | 4.96 years | NA |
|  |  |  |
| **Other medication used** |  |  |
| Aspirin | 182,390 (43.4%) | 8,617 (4.7%) |
| H2 blocker | 286,276 (68.1%) | 71,506 (39.2%) |
| Statin | 146,668 (34.9%) | 6,954 (3.8%) |
| NSAID | 223,953 (53.3%) | 77,763 (42.7%) |
| Anti-coagulant | 72,555 (17.3%) | 3,263 (1.8%) |
| Anti-platelet | 61,176 (14.5%) | 1,624 (0.9%) |
| PPI | 150,462 (35.8%) | 24,819 (13.6%) |
|  |  |  |
| **Disease diagnosis** |  |  |
| CHD/Stroke | 138,277 (32.9%) | 6,274 (3.4%) |
| CHD | 97,993 (23.3%) | 2,883 (1.6%) |
| Stroke | 60,518 (14.4%) | 3,603 (2.0%) |
| Diabetes mellitus | 141,625 (33.7%) | 9,781 (5.4%) |
|  |  |  |
| **Lung Cancer** | 11,029 (2.6%) | 6,623 (3.6%) |
|  |  |  |
| **Total death** | 158,348 (37.7%) | 39,817 (21.9%) |
|  |  |  |
| **Follow-up^*^** | 99.13 (48.6) | 140.8 (49.6) |

**Note**: ^*^ mean and standard deviation (SD).

**Abbreviations:** ACEi, angiotensin-converting enzyme inhibitor; ARB, angiotensin II receptor blocker; CCB, calcium channel blocker; H2, histamine-2 receptor; NSAID, non-steroidal anti-inflammatory drug; PPI, proton-pump inhibitor; CHD, coronary heart disease; NA, not applicable.

**Table S2.** Lung cancer risk assessment by the interaction effects analysis **^#^**

|  |  | **Anti-hypertensives *vs*  Non-Anti-hypertensives** | | | | | | |
| --- | --- | --- | --- | --- | --- | --- | --- | --- |
|  | **HR** | **95% CI** | ***p*** |  | ***p-_interaction_*** | **-2Log Likelihood** | ***p-_Likelihood_*** |  |
|  |  |  |  |  |  |  |  |  |
| **All Anti-Hypertensives** | **0.78** | **0.65-0.94** | **0.007** |  |  |  |  |  |
| **Aspirin** | 0.79 | 0.56-1.13 | 0.195 |  |  |  |  |  |
| Aspirin+Anti-hypertensives | **0.64** | **0.60-0.68** | **<0.001** |  |  |  |  |  |
| Aspirin* Anti-hypertensives |  |  |  |  | 0.090 | 423.723 | **< 0.001** |  |
|  |  |  |  |  |  |  |  |  |
| **Single Anti- Hypertensives** |  |  |  |  |  |  |  |  |
| **ACEi/ARB** | **0.79** | **0.68-0.91** | **0.043** |  |  |  |  |  |
| Aspirin+ACEi/ARB | 0.71 | 0.48-1.05 | 0.090 |  |  |  |  |  |
| ACEi/ARB*Aspirin |  |  |  |  | **0.024** | 213.925 | **<0.001** |  |
|  |  |  |  |  |  |  |  |  |
| ***β-*blocker** | **0.77** | **0.71-0.84** | **<0.001** |  |  |  |  |  |
| Aspirin+*β*-blocker | 0.81 | 0.62-1.005 | 0.115 |  |  |  |  |  |
| *β*-blocker *Aspirin |  |  |  |  | **0.035** | 215.598 | **<0.001** |  |
|  |  |  |  |  |  |  |  |  |
| **CCB** | **0.76** | **0.72-0.79** | **<0.001** |  |  |  |  |  |
| Aspirin+CCB | 0.82 | 0.64-1.06 | 0.129 |  |  |  |  |  |
| CCB*Aspirin |  |  |  |  | **0.049** | 247.247 | **<0.001** |  |
|  |  |  |  |  |  |  |  |  |
| ***α*-blocker** | 0.90 | 0.74-1.09 | 0.274 |  |  |  |  |  |
| Aspirin+*α*-blocker | **0.53** | **0.34-0.84** | **0.007** |  |  |  |  |  |
| *α*-blocker *Aspirin |  |  |  |  | **0.008** | 120.664 | **<0.001** |  |

**Note:** ^#^ Ref. group: Control; Age, sex, comorbidities, and medications were adjusted in the model.

**Abbreviations:** HR, hazard ratio; CI, confidence interval; ACEi, angiotensin converting enzyme inhibitor; ARB, angiotensin II receptor blocker; CCB, calcium channel blocker.

**Table S3.** Trend test for the follow-up analysis^*^

| **Duration/ Exposure drug** | **Anti-hypertensives only** | ***p*** | **Anti-hypertensives + Aspirin** | ***p*** |
| --- | --- | --- | --- | --- |
|  | **HR (95% CI)** |  | **HR (95% CI)** |  |
|  |  |  |  |  |
| **All Anti-hypertensives** |  |  |  |  |
| < 7 years | 1 |  | 1 |  |
| < 10 years | 0.23 (0.22-0.25) | <0.001 | 0.40 (0.37-0.43) | <0.001 |
| >=10 years | 0.10 (0.09-0.10) | <0.001 | 0.12 (0.11-0.13) | <0.001 |
| *P for trend^#^* |  | <0.001 |  | <0.001 |
|  |  |  |  |  |
| **ACEi/ARB** |  |  |  |  |
| < 7 years | 1 |  | 1 |  |
| < 10 years | 0.22 (0.10-0.49) | <0.001 | 0.37 (0.17-0.82) | 0.015 |
| >=10 years | 0.05 (0.01-0.34) | 0.003 | 0.23 (0.09-0.58) | 0.002 |
| *P for trend* |  | <0.001 |  | <0.001 |
|  |  |  |  |  |
| ***β*-blocker** |  |  |  |  |
| < 7 years | 1 |  | 1 |  |
| < 10 years | 0.38 (0.23-0.61) | <0.001 | 0.56 (0.35-0.90) | 0.017 |
| >=10 years | 0.08 (0.02-0.25) | <0.001 | 0.28 (0.18-0.45) | <0.001 |
| *P for trend* |  | <0.001 |  | <0.001 |
|  |  |  |  |  |
| **CCB** |  |  |  |  |
| < 7 years | 1 |  | 1 |  |
| < 10 years | 0.24 (0.18-0.31) | <0.001 | 0.32 (0.19-0.54) | <0.001 |
| >=10 years | 0.10 (0.06-0.16) | <0.001 | 0.29 (0.17-0.49) | <0.001 |
| *P for trend* |  | <0.001 |  | <0.001 |
|  |  |  |  |  |
| ***α*-blocker, male** |  |  |  |  |
| < 7 years | 1 |  | 1 |  |
| < 10 years | 0.34 (0.25-0.46) | <0.001 | 0.95 (0.46-1.92) | 0.871 |
| >=10 years | 0.22 (0.15-0.32) | <0.001 | 0.11 (0.02-0.83) | 0.032 |
| *P for trend* |  | <0.001 |  | 0.03 |

**Note:** ^*^ Ref. group: Control; Sex, comorbidities, and medications were adjusted in the model; ^#^ Linear trend for HR

**Abbreviations:** HR, hazard ratio; CI, confidence interval; ACEi, angiotensin-converting enzyme inhibitor; ARB, angiotensin II receptor blocker; CCB, calcium channel blocker

**Table S4.** Lung cancer risk of antihypertensive groups and combination of aspirin groups at different subgroups by age^*^

< 65 years old

| **Exposure** | **Anti-hypertensives** | | | |  | | **Anti-hypertensives + Aspirin** | | | | |  |
| --- | --- | --- | --- | --- | --- | --- | --- | --- | --- | --- | --- | --- |
|  | **N** | **Cases** | **HR (95% CI)** | ***p*** | |  | | **N** | **Cases** | **HR (95% CI)** | ***p*** | |
| **All Anti-hypertensives** | 46,400 | 613  (Control: 814) | 0.97 (0.85-1.12) | 0.712 | |  | | 55,434 | 937  (Control: 807) | 0.99 (0.86-1.14) | 0.883 | |
| **-ACEi/ARB** | 1,660 | 17  (Control: 184) | 0.78 (0.36-1.67) | 0.515 | |  | | 969 | 16  (Control:168) | 0.60 (0.29-1.23) | 0.160 | |
| ***-β*-blocker** | 3,073 | 27  (Control: 132) | 0.91 (0.56-1.48) | 0.705 | |  | | 3,583 | 45  (Control: 162) | 0.65 (0.39-1.08) | 0.099 | |
| **-CCB** | 3,416 | 32 (Control: 140) | 1.23 (0.67-2.25) | 0.666 | |  | | 1,202 | 23  (Control: 189) | 1.18 (0.65-2.15) | 0.588 | |
| ***-α*-blocker** | 2,381 | 26  (Control: 120) | 0.84 (0.52-1.35) | 0.468 | |  | | 335 | 4  (Control: 135) | 0.19 (0.02-2.11) | 0.179 | |

≥ 65 years old

| **Exposure** | **Anti-hypertensives** | | | |  | **Anti-hypertensives + Aspirin** | | | |  |
| --- | --- | --- | --- | --- | --- | --- | --- | --- | --- | --- |
|  | **N** | **Cases** | **HR (95% CI)** | ***p*** |  | **N** | **Cases** | **HR (95% CI)** | ***p*** | |
| **All Anti-hypertensives** | 191,673 | 5,979  (Control: 2,378) | 0.65 (0.61-0.68) | <0.001 |  | 127,229 | 3,533  (Control: 2,408) | 0.61 (0.57-0.65) | <0.001 | |
| **-ACEi/ARB** | 3,326 | 61  (Control: 471) | 0.42 (0.29-0.61) | <0.001 |  | 1,171 | 51  (Control:  469) | 0.84 (0.53-1.32) | 0.447 | |
| ***-β*-blocker** | 4,012 | 131  (Control: 482) | 0.76 (0.62-0.94) | 0.012 |  | 2,397 | 98 (Control:  525) | 0.85 (0.62-1.16) | 0.313 | |
| **-CCB** | 19,509 | 538  (Control: 478) | 0.90 (0.74-1.10) | 0.293 |  | 3,614 | 146  (Control: 475) | 0.83 (0.63-1.09) | 0.187 | |
| ***-α*-blocker** | 8,916 | 412  (Control: 318) | 0.57 (0.45-0.72) | <0.001 |  | 706 | 42  (Control: 319) | 0.69 (0.43-1.11) | 0.126 | |

**Note:** ^*^Ref. group: Control; Age, sex, comorbidities, and medications were adjusted in the model.

**Abbreviations:** N, number of participants; HR, hazard ratio; CI, confidence interval; ACEi, angiotensin-converting enzyme inhibitor; ARB, angiotensin II receptor blocker; CCB, calcium channel blocker

**Table S5.** Lung cancer risk of antihypertensive groups and combination of aspirin groups at different subgroups by sex^*^

Male

| **Exposure** | **Anti-hypertensives** | | | |  | | **Anti-hypertensives + Aspirin** | | | | | |
| --- | --- | --- | --- | --- | --- | --- | --- | --- | --- | --- | --- | --- |
|  | **N** | **Cases** | **HR (95% CI)** | ***p*** | |  | | **N** | **Cases** | **HR (95% CI)** | ***p*** |  |
| **All Anti-hypertensives** | 122,634 | 4,349  (Control: 2,229) | 0.58 (0.55-0.62) | <0.001 | |  | | 98,878 | 3,130  (Control: 2,304) | 0.59 (0.55-0.64) | <0.001 |  |
| **-ACEi/ARB** | 2,622 | 53  (Control: 482) | 0.44 (0.29-0.67) | <0.001 | |  | | 1,267 | 58  (Control: 465) | 0.76 (0.50-1.16) | 0.200 |  |
| ***-β*-blocker** | 2,692 | 83  (Control: 422) | 0.69 (0.53-0.90) | 0.006 | |  | | 3,157 | 97  (Control: 477) | 0.64 (0.46-0.91) | 0.012 |  |
| **-CCB** | 8,902 | 306  (Control: 442) | 0.79 (0.62-1.01) | 0.060 | |  | | 2,095 | 102  (Control: 465) | 0.80 (0.57-1.13) | 0.206 |  |
| ***-α*-blocker** | 11,297 | 438  (Control: 438) | 0.61 (0.48-0.77) | <0.001 | |  | | 1,041 | 46  (Control: 454) | 0.53 (0.34-0.84) | 0.007 |  |

Female

| **Exposure** | **Anti-hypertensives** | | | | | | |  | | **Anti-hypertensives + Aspirin** | | | | | | | | |
| --- | --- | --- | --- | --- | --- | --- | --- | --- | --- | --- | --- | --- | --- | --- | --- | --- | --- | --- |
|  | **N** | **Cases** | | **HR (95% CI)** | | ***p*** | |  | | **N** | | **Cases** | | **HR (95% CI)** | | ***p*** | |  |
| **All Anti-hypertensives** | 115,439 | 2,243  (Control: 963) | 0.74 (0.68-0.81) | | <0.001 | |  | | 83,785 | | 1,340  (Control: 911) | | 0.76 (0.67-0.85) | | <0.001 | |  |  |
| **-ACEi/ARB** | 2,364 | 25 (Control: 173) | 0.47 (0.27-0.82) | | 0.008 | |  | | 873 | | 9  (Control: 172) | | 0.45 (0.13-1.56) | | 0.207 | |  |  |
| ***-β*-blocker** | 4,393 | 75  (Control: 192) | 0.96 (0.71-1.29) | | 0.774 | |  | | 2,823 | | 46  (Control: 210) | | 1.22 (0.80-1.87) | | 0.357 | |  |  |
| **-CCB** | 14,023 | 264  (Control: 176) | 1.05 (0.76-1.46) | | 0.764 | |  | | 2,721 | | 67  (Control: 199) | | 0.86 (0.60-1.23) | | 0.398 | |  |  |

**Note:** ^*^Ref. group: Control; Age, sex, comorbidities, and medications were adjusted in the model.

**Abbreviations:** N, number of participants; HR, hazard ratio; CI, confidence interval; ACEi, angiotensin-converting enzyme inhibitor; ARB, angiotensin II receptor blocker; CCB, calcium channel blocker

**Table S6.** Lung cancer risk of antihypertensive groups and combination of aspirin groups at different subgroups by diabetes mellitus status^*^

Without DM

| **Exposure** | **Anti-hypertensives** | | | |  | | **Anti-hypertensives + Aspirin** | | | | | |
| --- | --- | --- | --- | --- | --- | --- | --- | --- | --- | --- | --- | --- |
|  | **N** | **Cases** | **HR (95% CI)** | ***p*** | |  | | **N** | **Cases** | **HR (95% CI)** | ***p*** |  |
| **All Anti-hypertensives** | 170,159 | 5,030  (Control: 2,962) | 0.67 (0.63-0.70) | <0.001 | |  | | 108,879 | 3,091  (Control: 2,975) | 0.67 (0.63-0.72) | <0.001 |  |
| **-ACEi/ARB** | 1,733 | 22  (Control: 608) | 0.40 (0.25-0.66) | <0.001 | |  | | 1,108 | 41  (Control: 594) | 0.54 (0.32-0.93) | 0.026 |  |
| ***-β*-blocker** | 6,316 | 129  (Control: 568) | 0.76 (0.61-0.94) | 0.012 | |  | | 4,924 | 114  (Control: 630) | 0.83 (0.62-1.10) | 0.189 |  |
| **-CCB** | 19,421 | 488  (Control: 576) | 0.88 (0.71-1.07) | 0.202 | |  | | 4,136 | 136  (Control: 622) | 0.80 (0.61-1.07) | 0.131 |  |
| ***-α*-blocker** | 10,314 | 398  (Control: 403) | 0.62 (0.49-0.80) | <0.001 | |  | | 868 | 37  (Control: 427) | 0.50 (0.30-0.82) | 0.007 |  |

With DM

| **Exposure** | **Anti-hypertensives** | | | |  | **Anti-hypertensives + Aspirin** | | | | |
| --- | --- | --- | --- | --- | --- | --- | --- | --- | --- | --- |
|  | **N** | **Cases** | **HR (95% CI)** | ***p*** |  | **N** | **Cases** | **HR (95% CI)** | ***p*** |  |
| **All Anti-hypertensives** | 67,914 | 1,562  (Control: 230) | 0.40 (0.35-0.47) | <0.001 |  | 73,784 | 1,379  (Control: 240) | 0.37 (0.32-0.44) | <0.001 |  |
| **-ACEi/ARB** | 3,253 | 56  (Control: 47) | 0.55 (0.35-0.85) | 0.007 |  | 1,032 | 26  (Control: 43) | 0.80 (0.45-1.42) | 0.450 |  |
| ***-β*-blocker** | 769 | 29  (Control: 46) | 1.01 (0.63-1.64) | 0.955 |  | 1,056 | 29  (Control: 57) | 0.62 (0.29-1.31) | 0.207 |  |
| **-CCB** | 3,504 | 82  (Control: 42) | 0.96 (0.54-1.74) | 0.905 |  | 680 | 33  (Control: 42) | 0.86 (0.50-1.48) | 0.584 |  |
| ***-α*-blocker** | 983 | 40  (Control: 35) | 0.47 (0.27-0.81) | 0.006 |  | 173 | 9  (Control: 27) | 0.82 (0.26-2.62) | 0.737 |  |

**Note:** ^*^Ref. group: Control; Age, sex, comorbidities, and medications were adjusted in the model.

**Abbreviations:** N, number of participants; HR, hazard ratio; CI, confidence interval; ACEi, angiotensin-converting enzyme inhibitor; ARB, angiotensin II receptor blocker; CCB, calcium channel blocker; DM, diabetes mellitus.

**Table S7.** Sensitivity analyses assessing the Lung cancer risk between antihypertensive groups and combination of aspirin groups by limited the minimum exposure period to 1, 1. 5, and 2 years from 6 months^*^

1 year

| **Exposure** | **Anti-hypertensives** | | |  | | **Anti-hypertensives + Aspirin** | | | | |
| --- | --- | --- | --- | --- | --- | --- | --- | --- | --- | --- |
|  | **Participants** | **HR (95% CI)** | ***p*** | |  | | **Participants** | **HR (95% CI)** | ***p*** |  |
| **All Anti-hypertensives** | 223,232 | 0.64 (0.61-0.68) | <0.001 | |  | | 176,935 | 0.67 (0.62-0.71) | <0.001 |  |
| **- ACEi/ARB** | 4,369 | 0.48 (0.33-0.70) | <0.001 | |  | | 1,908 | 0.63 (0.40-0.99) | 0.044 |  |
| ***-β*-blocker** | 6,073 | 0.81 (0.65-1.01) | 0.058 | |  | | 5,323 | 0.76 (0.56-1.02) | 0.065 |  |
| **-CCB** | 19,812 | 0.90 (0.75-1.09) | 0.292 | |  | | 4,249 | 0.84 (0.63-1.11) | 0.213 |  |
| ***-α*-blocker** | 9,703 | 0.64 (0.51-0.81) | <0.001 | |  | | 912 | 0.49 (0.28-0.83) | 0.009 |  |

1.5 year

| **Exposure** | **Anti-hypertensives** | | |  | | **Anti-hypertensives + Aspirin** | | | |
| --- | --- | --- | --- | --- | --- | --- | --- | --- | --- |
|  | **Participants** | **HR (95% CI)** | ***p*** | |  | | **Participants** | **HR (95% CI)** | ***p*** |
| **All Anti-hypertensives** | 211,392 | 0.67 (0.64-0.71) | <0.001 | |  | | 171,641 | 0.71 (0.66-0.76) | <0.001 |
| **-ACEi/ARB** | 4,010 | 0.49 (0.33-0.73) | <0.001 | |  | | 1,750 | 0.46 (0.28-0.77) | 0.003 |
| ***-β*-blocker** | 5,483 | 0.89 (0.71-1.12) | 0.328 | |  | | 4,937 | 0.69 (0.49-0.96) | 0.028 |
| **-CCB** | 17,790 | 0.96 (0.79-1.16) | 0.663 | |  | | 3,900 | 0.91 (0.67-1.25) | 0.568 |
| ***-α*-blocker** | 8,729 | 0.63 (0.50-0.81) | <0.001 | |  | | 816 | 0.47 (0.26-0.84) | 0.011 |

2 years

| **Exposure** | **Anti-hypertensives** | | |  | | **Anti-hypertensives + Aspirin** | | | |
| --- | --- | --- | --- | --- | --- | --- | --- | --- | --- |
|  | **Participants** | **HR (95% CI)** | ***p*** | |  | | **Participants** | **HR (95% CI)** | ***p*** |
| **All Anti-hypertensives** | 200,369 | 0.70 (0.66-0.74) | <0.001 | |  | | 166,283 | 0.76 (0.70-0.81) | <0.001 |
| **-ACEi/ARB** | 3,631 | 0.52 (0.33-0.82) | 0.005 | |  | | 1,599 | 0.55 (0.32-0.94) | 0.030 |
| ***- β*-blocker** | 5,032 | 0.95 (0.75-1.21) | 0.692 | |  | | 4,654 | 0.79 (0.56-1.11) | 0.176 |
| **-CCB** | 15,827 | 0.98 (0.81-1.20) | 0.871 | |  | | 3,578 | 0.98 (0.71-1.36) | 0.909 |
| ***-α*-blocker** | 7,812 | 0.64 (0.50-0.82) | <0.001 | |  | | 725 | 0.42 (0.22-0.82) | 0.011 |

**Note:** ^*^ Ref. group: Control; Age, sex, comorbidities, and medications were adjusted in the model

**Abbreviations:** HR, hazard ratio; CI, confidence interval; ACEi, angiotensin-converting enzyme inhibitor; ARB, angiotensin II receptor blocker; CCB, calcium channel blocker
